# Predicting post-trauma stress symptoms from pre-trauma psychophysiologic reactivity, personality traits and measures of psychopathology

**DOI:** 10.1186/2045-5380-2-8

**Published:** 2012-05-18

**Authors:** Scott P Orr, Natasha B Lasko, Michael L Macklin, Suzanne L Pineles, Yuchiao Chang, Roger K Pitman

**Affiliations:** 1 Department of Psychiatry, Massachusetts General Hospital and Harvard Medical School, Charlestown, MA, USA; 2 Department of Veterans Affairs Medical Center, Manchester, NH, USA; 3 National Center for PTSD, Women’s Health Sciences Division, VA Boston Healthcare System, Boston, MA, USA; 4 Department of Psychiatry, Boston University School of Medicine, Boston, MA, USA; 5 General Medicine Division, Massachusetts General Hospital and Harvard Medical School, Boston, MA, USA

**Keywords:** Stress disorders, Post-traumatic, Conditioning, Startle, Imagery, Psychophysiology, Risk factors

## Abstract

**Background:**

Most individuals exposed to a traumatic event do not develop post-traumatic stress disorder (PTSD), although many individuals may experience sub-clinical levels of post-traumatic stress symptoms (PTSS). There are notable individual differences in the presence and severity of PTSS among individuals who report seemingly comparable traumatic events. Individual differences in PTSS following exposure to traumatic events could be influenced by pre-trauma vulnerabilities for developing PTSS/PTSD.

**Methods:**

Pre-trauma psychological, psychophysiological and personality variables were prospectively assessed for their predictive relationships with post-traumatic stress symptoms (PTSS). Police and firefighter trainees were tested at the start of their professional training (i.e., pre-trauma; n = 211) and again several months after exposure to a potentially traumatic event (i.e., post-trauma, n = 99). Pre-trauma assessments included diagnostic interviews, psychological and personality measures and two psychophysiological assessment procedures. The psychophysiological assessments measured psychophysiologic reactivity to loud tones and the acquisition and extinction of a conditioned fear response. Post-trauma assessment included a measure of psychophysiologic reactivity during recollection of the traumatic event using a script-driven imagery task.

**Results:**

Logistic stepwise regression identified the combination of lower IQ, higher depression score and poorer extinction of forehead (corrugator) electromyogram responses as pre-trauma predictors of higher PTSS. The combination of lower IQ and increased skin conductance (SC) reactivity to loud tones were identified as pre-trauma predictors of higher post-trauma psychophysiologic reactivity during recollection of the traumatic event. A univariate relationship was also observed between pre-trauma heart rate (HR) reactivity to fear cues during conditioning and post-trauma psychophysiologic reactivity.

**Conclusion:**

The current study contributes to a very limited literature reporting results from truly *prospective* examinations of pre-trauma physiologic, psychologic, and demographic predictors of PTSS. Findings that combinations of lower estimated IQ, greater depression symptoms, a larger differential corrugator EMG response during extinction and larger SC responses to loud tones significantly predicted higher PTSS suggests that the process(es) underlying these traits contribute to the pathogenesis of subjective and physiological PTSS. Due to the low levels of PTSS severity and relatively restricted ranges of outcome scores due to the healthy nature of the participants, results may underestimate actual predictive relationships.

## Background

Most individuals exposed to a traumatic event do not develop post-traumatic stress disorder (PTSD); however, many individuals may experience varying amounts of sub-clinical levels of post-traumatic stress symptoms (PTSS). Notable differences in the presence and severity of PTSS have been found among individuals reporting traumatic events that would appear to be comparably stressful [1-3]. This raises the possibility that some individuals may have pre-trauma vulnerabilities for developing PTSS or diagnosable PTSD.

Increased vulnerability to PTSS has been found to be associated with individual difference factors such as: personal history of depressive disorders [4,5], anxiety disorders [4] as well as introversion [6] and neuroticism [4,6,7]. Underlying these various vulnerabilities may be a common factor termed, “negative affectivity.” Clark, Watson and Mineka [8] have described negative affectivity as a “…stable, heritable, and highly general trait dimension with a multiplicity of aspects ranging from mood to behavior” that is “…broadly related to psychopathology, including indices of both anxiety and depression…” (p. 104). Thus, negative affectivity would encompass a range of characteristics demonstrated to be highly correlated with each other and potentially with PTSS, including: anxiety, introversion, neuroticism, electrodermal lability, slowed habituation rate and increased conditionability [6,7,9].

Numerous studies have demonstrated concurrent associations between psychologic and psychophysiologic factors and PTSD symptoms. This research has primarily used cross-sectional study designs, i.e., comparisons are made between individuals with and without PTSD. From such studies it is difficult, if not impossible, to determine whether the observed differences are a consequence of developing PTSD or might represent pre-existing differences that are associated with risk for developing PTSD. We examined the PTSD literature to identify well-standardized psychologic and psychophysiologic measures that have been found to discriminate between individuals with and without PTSD in at least one or more published studies and that might serve as risk markers for the development of PTSD. The candidate variables we selected from this review as potential risk markers, in addition to age, education, gender and race, are listed in Table 1.

**Table 1:** Pre-trauma Predictor Variable Means and SDs for Individuals Exposed to a Traumatic Event (N = 99)

**Variable**	**M**	**SD**
Psychometric Measures		
SCL-90-R GSI	0.3	0.4
BDI	2.5	3.2
WAIS-R EST-IQ	101.8	10.0
STAI-T	31.2	8.2
NEO-PI-R Domain		
Neuroticism	46.7	8.0
Extroversion	54.7	8.2
Openness	48.0	11.3
Agreeableness	48.0	9.4
Loud-tone Psychophysiologic Measures		
Heart rate		
Pre-tone level (bpm)	71.6	11.6
Mean response (√bpm)	0.6	0.9
Response slope	0.1	1.0
Skin conductance		
Pre tone level (μS)	7.2	5.6
Mean response (√μS)	0.6	0.3
Response slope	-0.4	0.2
Trials to criterion	9.7	4.2
Electromyogram		
Mean response (√μV)	2.3	1.7
Fear-conditioning Psychophysiologic Measures		
SC Orienting Response	0.6	0.7
Differential Response		
Acquisition Phase		
SC (μS)	0.2	0.5
EMG (μv)	0.2	2.9
HR (bpm)	1.0	5.7
Extinction Phase		
SC (μS)	-0.1	0.5
EMG (μV)	-0.7	4.4
Response to CS + trials		
Acquisition Phase		
SC (μS)	0.6	0.6
EMG (μv)	2.5	3.0
HR (bpm)	5.8	4.8
Extinction Phase		
SC (μS)	0.2	0.4
EMG (μV)	3.1	4.2

Note: SCL-90-R GSI = Symptom Checklist 90-Revised, Global Severity Score; BDI = Beck Depression Inventory; WAIS-R EST-IQ = Estimated WAIS-R Full Scale IQ from Shipley Institute of Living Scale; STAI-S = State/Trait Anxiety Inventory-State score; bpm = beats per minute; S = Siemens; V = volts; SC = skin conductance; HR = heart rate; Orienting Response = mean response to first presentation of the CS + and the CS- during the habituation phase; Acquisition and extinction response to CS + trials = mean response to CS + trials during the acquisition and extinction phases, respectively; habituation, acquisition, and extinction differential responses = the mean CS interval response to CS + trials minus the mean CS interval response to CS- trials for the habituation, acquisition, and extinction phases, respectively; CS = conditioned stimulus.

Briefly, studies of psychologic measures have found individuals with, compared to without, PTSD to have greater: general psychiatric symptoms e.g., [10-13], depression symptoms e.g., [11,12,14-16], state and/or trait anxiety e.g., [11-15,17-19] and neuroticism e.g., [16,20-23]. Whereas, greater: IQ [16,24-27], extraversion [22,28], openness [29] and agreeableness [22,23] have been observed in individuals without, compared to with, PTSD. Of particular note, research cited above suggests that lower IQ and the personality traits of increased neuroticism, lower extraversion and lower agreeableness are pre-trauma predictors of increased risk for PTSD and/or PTSS.

Studies of psychophysiologic reactivity to startling stimuli and fear conditioning have reported several reliable differences between individuals with and without PTSD. The startle response represents a form of unconditioned reactivity to sudden, unexpected stimuli such as a loud noise that is mediated by brainstem neural circuitry [30] but is influenced by higher psychological processes such as emotional state. Magnitude of the eyeblink response is commonly used as the prototypical measure of startle in humans. However, startling acoustic stimuli can also produce increased skin conductance (SC) and heart rate (HR) responses, indicating that there are autonomic, as well as muscular, features of startle. The magnitude of reactivity to startling stimuli and rate of decline in response magnitude over repeated presentations of a startle stimulus, i.e., habituation, have been extensively used as measures of anxiety and/or emotional state. There is a substantial literature demonstrating larger magnitude: eyeblink e.g., [17,31,32], skin conductance e.g., [19,32,33] and heart rate e.g., [10,17,19,31,33-36] responses to loud noise or tone stimuli in individuals with, compared to without, PTSD. Of particular note, findings from a recent prospective study of firefighter trainees suggests that eyeblink and SC response magnitudes to loud tones predict the development of greater PTSS following exposure to a traumatic event [37]. In contrast, there is now compelling evidence that larger HR response magnitude to loud tones is an acquired feature of PTSD [36,38]. Slower habituation of SC responses e.g., [10,17,33,35,36], HR responses [34] and eyeblink responses [38] have also been observed in individuals with PTSD. There is some evidence that slower habituation of SC and eyeblink responses, as measured by the number of trials to no-response, may be acquired features of PTSD [38].

Psychophysiologic studies of acquisition and extinction of conditioned fear responses have also demonstrated differences between individuals with and without PTSD. Fear conditioning provides a measure of an individual's propensity to associate a neutral cue with an aversive outcome. This propensity can be assessed from the strength of the conditioned response that is established during acquisition, as well as from its resistance to extinction. Measures of SC activity have been commonly used to assess human fear conditioning; measures of HR and facial EMG activity have been less frequently used. Over the past three decades, differential fear conditioning, a procedure that pairs one of two neutral stimuli with a mildly aversive unconditioned stimulus (UCS), has been widely used to study conditioned fear in humans. This conditioning procedure allows for the calculation of differential response scores, calculated as the average response to the conditioned stimulus (CS+) paired with the UCS minus the average response to the stimulus (CS-) not paired with the UCS. These scores reflect the amount of conditioned responding to the fear cue (CS+) during acquisition and extinction. Individuals with PTSD have been found to show larger differential SC [11,14], corrugator EMG [11] and HR [11,18] responses during acquisition and larger SC [11,14,39] responses during extinction. Of particular relevance to the present work is the recent finding from a study of firefighter trainees that the magnitude of the differential corrugator EMG response during acquisition and extinction was significantly correlated with PTSS following exposure to a traumatic event [40].

To date, only a very few studies have addressed whether the aforementioned psychologic and psychophysiologic measures represent pre-trauma vulnerability markers or are consequences of developing PTSD/PTSS. From a practical standpoint, the identification of vulnerability markers for PTSD could have important practical implications related to screening and targeting early intervention for at-risk individuals working in high-risk occupations. From a theoretical standpoint, addressing whether or not psychologic and psychophysiologic features associated with PTSS represent pre-trauma or acquired traits has substantial implications for understanding the pathogenesis of PTSS and PTSD.

The work reported here assessed pre-trauma psychologic and psychophysiologic measures in order to evaluate their ability to predict the development of PTSD/PTSS severity following exposure to an intervening traumatic event. In order to increase the likelihood of finding participants who would be exposed to a traumatic event at some proximate point following pre-trauma assessment, we recruited firefighter and police trainees at the time of their initial training. The pre-trauma assessment included structured diagnostic interviews, standard psychometrics, and psychophysiologic reactivity to loud-tones and differential fear conditioning. Participants were closely followed for the occurrence of a traumatic event that met the DSM-IV PTSD Category A1 criterion during the course of their new professional duties. After such a traumatic event, the participant was invited to return for psychodiagnostic, psychometric and psychophysiologic assessments. As part of the post-trauma assessment, psychophysiologic reactivity to individually-tailored imagery scripts was measured as a non-subjective measure of emotional reactivity to the traumatic event.

The primary goal of the work reported here was to prospectively identify the best combination of psychologic and psychophysiologic variables that predicted PTSD/PTSS following exposure to a potentially traumatic event. Based upon the sparse but most relevant literature mentioned above, we hypothesized that individuals with higher PTSD/PTSS following exposure to a traumatic event would show the following pre-trauma traits: 1) greater conditionability (i.e., stronger acquisition and slower extinction of an aversively conditioned differential corrugator EMG response); 2) larger magnitude eyeblink EMG and SC responses to loud tones; 3) a higher mean SC level; 4) lower IQ; and 5) lower extraversion, higher neuroticism, and lower agreeableness scores on the Revised NEO Personality Inventory [41].

## Methods

### Participants

Three hundred eight firefighter/EMT and police trainees initially provided written informed consent to participate, which was obtained from all participants in accordance with the requirements of the Human Research Committee at the Massachusetts General Hospital and then completed the initial diagnostic, psychometric and psychophysiologic assessments. Firefighter trainees were recruited from the Lakes Region Community College, Laconia, NH and Fire Academy, Boston Fire Department, Boston, MA. Police trainees were recruited from the Lowell Police Academy, Lowell, MA. Ninety-nine participants reached the project’s endpoint, which required exposure to a traumatic event and follow-up testing consisting of diagnostic, psychometric and psychophysiologic assessments. Firefighters comprised 64.9 percent of the sample and the majority was male (91.8 %) and Caucasian (82.2 %). Mean age and education level were 27.0 (SD = 6.5) and 14.1 (SD = 1.8) years, respectively. The average participant fell within the normal range of IQ (Estimated WAIS-R Full Scale IQ: M = 101.8, SD = 10.0) as determined from the Shipley Institute of Living Scale [42,43] and was mentally healthy as measured by depression (Beck Depression Inventory: M = 2.5, SD = 3.2); general psychiatric symptoms (Symptom Checklist-90-Revised Global Severity Index (GSI): M = 0.3, SD = 0.4) and trait anxiety (Speilberger State-Trait Anxiety Inventory: M = 31.2, SD = 8.2). The Structured Clinical Interview for DSM-IV (SCID, [44]) and Clinician-Administered PTSD Scale (CAPS, [45]) were conducted during the pre-trauma assessments at the respective training academies. As determined from the SCID, the percentages of individuals who met DSM-IV criteria for a current diagnosis were as follows: alcohol dependence = 2 %, substance dependence < 1 %, major depression < 1 %, ADHD = 2 %, phobia < 1 %. The percentages of individuals who met DSM-IV criteria for a lifetime diagnosis were as follows: alcohol dependence = 10 %, substance dependence = 3 %, major depression = 3 %, and panic disorder < 1 %. As determined from the CAPS, n = 6 individuals met DSM-IV criteria for a current or lifetime diagnosis of PTSD at the time of the initial assessment and were excluded from the reported study. Trainees who completed the Stressful Life Events Checklist (n = 212) reported experiencing an average of 1.6 (SD = 1.5) different types of traumatic events before the initial assessment, with first exposure at an average age of 10.9 (SD = 8.4) years.

### Measures and Procedures

#### ***Pre-trauma diagnostic assessment***

The SCID Screen Patient Questionnaire (SSPQ) Computer Program for DSM-IV [46] and SCID Overview were administered to each trainee. If a trainee answered yes to any SSPQ items, a diagnostician administered the appropriate SCID module. If the trainee endorsed items that indicated that a traumatic event had occurred and that PTSD-like symptoms were experienced, the CAPS was administered in order to determine whether the participant had current and/or past PTSD and to obtain a continuous score corresponding to overall PTSS/PTSD severity (CAPS total score).

#### ***Pre-trauma psychometric assessment***

The following instruments were administered: 1) Stressful Life Events Checklist [47], which solicits the frequency of previous traumatic event(s) experienced; 2) NEO-PI-R [41], which measures the dimensions of neuroticism, extraversion, openness to experience, agreeableness, and conscientiousness; 3) SCL-90-R [48] ; 4) STAI [49]; 5) BDI [50]; and 6) Shipley Institute of Living Scale [43], which estimates WAIS-R IQ.

#### ***Pre-trauma psychophysiologic assessment***

Left orbicularis oculi and corrugator EMG, SC, and HR were recorded using a Coulbourn Lablinc V-series modular instrument system (Coulbourn Instruments LLC, Whitehall, PA). EMG signals were amplified, filtered to include the 90 to 1000 Hz range, and integrated using a 10-msec time constant for orbicularis oculi and a 200-msec time constant for corrugator EMG. For EMG, the skin was abraded and 4-mm (sensor diameter) Invivo Metric Ag/AgCl electrodes filled with electrolyte paste were placed over the left orbicularis oculi and corrugator muscle sites according to published specifications [51]. Skin conductance level was measured directly using a constant 0.5 V through 9-mm (sensor diameter) Invivo Metric Ag/AgCl electrodes placed on the hypothenar surface of the participant's non-dominant hand in accordance with published guidelines [52]. Heart rate was measured through 9-mm (sensor diameter) Invivo Metric Ag/AgCl electrodes filled with electrolytic paste and placed on the inside of each arm; HR was converted from interbeat interval. All psychophysiologic analog signals were digitized at 1000 Hz.

##### *Loud-tones procedure*

An audiologic screening test was performed to verify that participants could hear a 1000-Hz tone at the 25 dB level in each ear. The stimuli, laboratory assessment procedure and dependent psychophysiologic measures (left orbicularis oculi, SC, & HR) were the same as used in several previous studies of PTSD [17]. The stimuli consisted of 15, 95-dB (sound pressure level), 1000-Hz pure tones presented for 500-ms, with 0-ms rise and fall times generated by a Coulbourn Audio Source Module (V85-05). Stimuli were presented binaurally over headphones. Intertrial intervals were randomly chosen to range between 27 and 52 s.

The psychophysiologic assessment session took place in a humidity- and temperature-controlled room located in quiet areas of the respective training academies. Due to space limitations, the participant, laboratory equipment and technician were located in the same room. A screen prevented the participant from seeing the recording equipment and technician during the testing procedures. The participant was seated in a comfortable chair and, after the recording electrodes had been attached, participants were read the standard instructions as used in our previous research [17]. The technician then placed headphones on the participant, checked that the physiologic measures were being properly recorded, and activated the computer. Following a 5-min resting, baseline-recording period, the series of 15 tones was presented.

##### *Conditioning procedure*

The conditioning procedure took place after the loud-tone task was completed. The participant was seated approximately 4 feet in front of a monitor that was used to display the CSs. The CS + and CS- were represented by 6-inch diameter blue and white circles, respectively. The UCS was a 500-ms electric shock generated by a Coulbourn Transcutaneous Aversive Finger Stimulator (E13-22) and previously selected by the participant to be "highly annoying but not painful," delivered through electrodes attached to the first and third fingers of the dominant hand. Once the UCS level had been selected, the trainee was given the same instructions as used by [11].

After a 5-min resting period, the three phases of the experiment were initiated. Habituation (Phase I) consisted of five similar presentations each of the to-be CS + and CS- in pseudo-random order, i.e., no more than two consecutive presentations of the same stimulus type. The CS duration was 8 s, with randomly determined intertrial intervals ranging from 15–25 s. Acquisition (Phase II) consisted of five presentations of each stimulus type; a 500-ms shock pulse occurred immediately following each CS + offset. Extinction (Phase III) consisted of 10 similar, non-reinforced presentations each of the CS + and CS-. In each of these three phases, SC, HR, and left corrugator EMG were sampled beginning 4 s prior to CS onset and ending 6 s following CS offset.

#### ***Monitoring for a traumatic event***

Each trainee who completed the pre-trauma assessment was contacted by mail on a bi-monthly basis to inquire as to the occurrence of a traumatic event during the previous two months that potentially met the DSM-IV PTSD Category A1 criterion. It was not required that an individual also meet the A2 criterion. Those individuals who reported such an “index” event were invited for the post-trauma assessment. The traumatic experiences included: witnessing death or severe injury (n = 59), threat to one's physical integrity (e.g., explosions, fires, high-speed chase, n = 16), motor vehicle accident (n = 4), physical assault (n = 4), and death or serious injury to someone known to the participant (n = 16). The mean amount of time elapsed between the pre-trauma assessment and the reported traumatic event was 19.4 months (SD = 12.2), and between the traumatic event and post-trauma assessment was 12.3 months (SD = 11.5).

#### ***Post-trauma assessment***

This consisted of two visits separated by approximately one week, the first for psychodiagnostic and psychometric evaluations, and the second for laboratory testing. The CAPS was re-administered during the first visit and was focused on the index traumatic event, and the SCID was re-administered to assess for any new non-PTSD DSM-IV Axis I mental disorders. Participants also completed the Impact of Event Scale-Revised (IES-R, [53]). Script preparation for the script-driven imagery procedure, which took place the following week, was conducted according to our published procedure [1].

Prior to the laboratory testing during the second visit, a urine sample was obtained for drug screening; results indicated negligible drug use.

##### *Psychophysiologic reactivity during script-driven imagery*

Results from more than 20 studies have provided consistent evidence of heightened psychophysiologic reactivity (SC, HR, or facial EMG responses) to cues reminiscent of a traumatic event in individuals with, compared to without, a diagnosis of current PTSD [54]. Assessment of psychophysiologic responses during imagery of the index traumatic event and other life events was performed according to our published procedure [1]. Personalized “scripts” approximately 30 s in length, composed in the second person, present tense, were created portraying each individual’s traumatic events. Scripts related to other types of personal experiences, including stressful, positive, and neutral experiences, were also created. Standard scripts portraying various hypothetical experiences were also used. Scripts were recorded and played back to participants while they vividly imagined each event; HR, SC and facial EMG (left lateral frontalis and corrugator) were measured throughout.

Transcripts of the two personalized traumatic scripts for each individual were reviewed in scrambled order by a highly experienced clinical psychologist who treats patients with PTSD. The clinician rated each script on a 0 – 12 Likert-type scale for the severity of the traumatic event with “12” indicating “the most traumatic event you have encountered in your clinical experience,” as we have done previously [2,55]. Ratings of the two traumatic scripts were averaged for each individual to provide a measure of the apparent severity of the traumatic exposure. The average severity rating was M = 7.79, SD = 1.41.

### Data reduction

#### ***Psychophysiologic response scores for the loud-tone procedure***

Each subject's psychophysiological records were reviewed by eye and statistically for aberrant individual response scores. Where a response was clearly aberrant (e.g., a doubling of HR caused by detection of the EKG T-wave), the aberrant value was replaced by the average of responses to the immediately preceding and following trials. We estimate that less than 5 % of data were unusable or imputed. Psychophysiologic responses to the loud tones were quantified in the same manner as in our previous work. An eyeblink EMG response was calculated for each trial by subtracting the average orbicularis oculi level during the 1-s interval immediately preceding tone onset from the highest orbicularis oculi value within 40–200 ms following tone onset. A SC response was calculated for each tone trial by subtracting the average SC level during the 1 s immediately preceding tone onset from the highest SC value within 1–4 s following tone onset. An accelerative HR response was calculated for each tone trial by subtracting the average pre-stimulus HR, as determined from the two heart beats immediately preceding tone onset, from the highest HR value (i.e., shortest IBI) within 1–4 s following tone onset. A square-root transformation was applied to all responses to reduce skewness and heteroscedasticity. Response scores included the mean HR, SC, and eyeblink EMG responses (averaged across the 15 tones). Habituation of responses was measured in two ways. Absolute habituation of SC and EMG responses was assessed by counting the number of trials before reaching a criterion of two successive non-response trials (SC < .05 μS; EMG < .30 μV). Relative habituation was calculated for all measures from the slope of the regression equation *Y = bx + a* for Trials 2–15, whereby Y is the square root of the response score, and x is the log trial number.

#### ***Psychophysiologic response scores for aversive conditioning***

A response score for each acquisition and extinction trial for each measure was calculated by subtracting the average SC, corrugator EMG and HR level for the 2-s interval immediately preceding CS onset from the respective measure’s highest value during the 8-s CS interval, as has been previously done [11,56]. “Conditionability” scores for the acquisition and extinction phases were calculated by subtracting the mean SC, corrugator EMG, and HR responses to CS- trials from the respective mean responses to CS + trials. This was done separately for the acquisition and extinction phase.

In addition to examining differential conditioning scores, we included measures that represented SC, corrugator EMG and HR reactivity to only CS + trials during acquisition and extinction. There is evidence suggesting that some individuals with PTSD, while responding strongly to the CS+, respond equally strongly to the CS- thereby producing a small differential conditioning score [e.g., [18]. Thus, a measure that only reflects reactivity to CS + trials might provide a better estimate of reactivity to fear cues. An unconditioned response (UCR) score was calculated by subtracting the average level for the 2-s interval immediately preceding UCS onset from the highest value among those recorded during the UCS interval (0–6 s post-UCS offset).

#### ***Psychophysiologic response scores for script-driven imagery***

A response score was calculated for each psychophysiologic dependent variable, separately for the two trauma-related scripts, by subtracting the preceding average baseline period value from the average value during imagery. The respective psychophysiologic response scores were then averaged for the two trauma-related scripts. Next, a single index of psychophysiologic reactivity to trauma-related script-driven imagery was obtained using an a priori discriminant function. This function was previously derived using discriminant analysis, a procedure that mathematically determined the optimal weightings for the combination of HR, SC, and lateral frontalis EMG responses during trauma-related imagery of previously studied individuals that maximally distinguished those with versus without current PTSD [1-3,55,57,58]. The sensitivity and specificity of this function are 57 % and 89 %, respectively [59]. Once derived, the discriminant function can be applied to a given individual's HR, SC, and lateral frontalis EMG responses to trauma-related imagery to yield a single score, i.e., “posterior probability” of having PTSD. For the present study, this *a priori* discriminant function was applied to each participant's respective HR, SC, and lateral frontalis EMG responses to trauma-related imagery, thereby providing a single measure of PTSS based solely on their psychophysiological reactivity to trauma-related imagery that can be viewed as an objective measure of emotional reactivity.

### Statistical analysis

Variables were summarized using means and SDs or percentages. Study participants who reported experiencing a traumatic event were invited to return to our laboratory to complete the post-trauma assessments. The primary psychologic outcome measures of the study included the CAPS and IES-R total scores. Because no subject met full criteria for PTSD and CAPS total scores were very low (M = 4.1, SD = 8.3, range = 0.0–41.0), i.e., well within the normal range, the IES-R was used as the measure of PTSS severity, as has been done in previous prospective studies that also failed to find cases of PTSD [37,40]. The primary psychophysiologic outcome measure was the posterior probability score derived from the script-driven imagery assessment. Distributions of scores on the outcome measures were highly skewed due to participants’ low symptom severity levels. For this reason we dichotomized scores into “high” (upper tertile) and “low” (lower two tertiles) groups to avoid the use of variable transformation and any linearity assumption. The lower two tertiles were combined because scores in the first and second tertiles were comparably low. In order to examine the robustness of results using the aforementioned dichotomization of scores within the current sample, the outcome variables were also tested after being dichotomized into the highest (Q4) and lowest (Q1) quartile groups, as well as into the highest (T3) and lowest (T1) tertile groups, so as to maximize differences between the groups. Results from the three dichotomization strategies were very similar. Consequently, we chose to create the “low” group from the combination of the lower two tertiles (T2, T3) because this strategy used all available data, whereas the other two strategies used only half (Q4 vs. Q1) or two-thirds (T3 vs. T1) of the data.

The list of psychometric, and psychophysiologic pre-trauma predictor variables is presented in Table 1 and, as noted above, were selected primarily on the basis of their having previously been found to discriminate between individuals with and without PTSD. The demographic variables, gender, race, age and education level, were also included as potential predictor variables. Separate logistic stepwise regression analysis using the predictor variables that were found to demonstrate a difference at p < .20 for the dichotomized IES-R scores and dichotomized Posterior Probability scores, respectively, were performed. For the regression models, the missing values from the potential predictors were imputed from other available predictor values using the EM algorithm [60] to preserve the sample size.

## Results

### Relationships between pre-trauma predictor variables and IES-R scores in trauma-exposed individuals

#### ***Post-trauma outcome measures***

The IES-R served as the primary outcome measure; the overall mean and SD for IES-R scores was: M = 4.8, SD = 9.1, range = 0.0–46.5 and Posterior Probability score (psychophysiologic reactivity during trauma-related imagery), M = 0.35, SD = 0.11, range = 0.15–0.95).

#### ***Univariate comparisons for pre-trauma predictors of post-trauma outcome***

Relationships between pre-trauma predictors (listed in Table 1) and post-trauma IES-R and Posterior Probability scores (dichotomized as High vs. Low) were examined by means of t-test for unequal variances, a more conservative test than that for equal variances. The following pre-trauma measures produced scores that were significantly different (or nearly so) between the High vs. Low IES-R groups: BDI-II (High: M = 3.6, SD = 4.0; Low: M = 1.9, SD = 2.7; t(46) = 2.17, p = .035), Shipley Estimated IQ (High: M = 98.4, SD = 13.2; Low: M = 103.5, SD = 7.7; t(42) = 2.00, p = .052), STAI-Trait (High: M = 33.7, SD = 18.3; Low: M = 29.8, SD = 8.0; t(60) = 2.17, p = .034), differential corrugator EMG response scores during extinction (High: M = 0.6, SD = 5.1; Low: M = −1.5, SD = 4.0; t(51) = 2.07, p = .043).

The following pre-trauma measures produced scores that were significantly different (or nearly so) between the High vs. Low Posterior Probability score groups: Shipley Estimated IQ (High: M = 98.4, SD = 11.7; Low: M = 103.9, SD = 7.8; t(43) = 2.36, p = .023), mean SC response to loud tones (High: M = 0.7, SD = 0.7; Low: M = 0.5, SD = 0.3; t(53) = 1.99, p = .052), number of trials to reach the SC habituation non-response criterion (High: M = 10.9, SD = 4.1; Low: M = 9.2, SD = 4.2; t(61) = 1.87, p = .066), mean HR response to CS + trials during acquisition (High: M = 7.1, SD = 3.8; Low: M = 4.9, SD = 4.5; t(75) = 2.10, p = .039).

#### ***Combined pre-trauma predictors of post-trauma outcome***

Results of stepwise logistic regression analyses predicting IES-R and Posterior Probability score High vs. Low groups are presented in Table 2. As can be seen in the table, logistic regression identified lower estimated IQ, higher pre-trauma Beck Depression Inventory-II score and a larger differential corrugator EMG response during extinction as the best combination of predictor variables for higher post-trauma IES-R scores. The corresponding c-statistic for the IES-R model derived from the maximized sample was 0.70, indicating that 70 percent of individuals with high IES-R scores were correctly classified, suggesting reasonably good model performance. In order to examine the potential impact of individual differences in the amount of time elapsed between occurrence of the traumatic event and subsequent laboratory testing and in the clinician-rated severities of the traumatic events, logistic regression analyses were repeated with the amount of time-since-trauma and trauma-severity ratings added to the models. Odds ratios were 1.02 for the time-since-trauma variable and 0.90 for the trauma-severity variable when they were entered into the model; their contributions to the model were non-significant (Wald Chi-Square (1) = 0.84, p = .359 and Wald Chi-Square (1) = 0.33, p = .564, respectively).

**Table 2:** Results of Multiple Logistic Stepwise Regression Predicting Post-trauma Outcome from the Set of Pre-trauma Variables

**Maximized Sample**						
Outcome		Selected	Odds	Wald		
Measure	N	Predictor(s)	Ratio	Chi-Square	df	p
IES-R	96	Shipley Est. IQ	0.95	4.28	1	.039
		EMG Diff_E1	1.17	4.46	1	.035
		BDI-II	1.15	3.50	1	.062
Post. Prob.	95	Shipley Est. IQ	0.94	5.72	1	.017
		SC Mean Resp.	4.02	3.70	1	.055

Note: Post. Prob. = Posterior Probability score derived from script-driven imagery assessment; IES-R = Impact of Event Scale-Revised total score; Shipley Est. IQ = Estimated WAIS-R Full Scale IQ from Shipley Institute of Living Scale; EMG Diff_E1 = corrugator electromyogram differential response during extinction, i.e., the averaged CS interval response to the first five CS + trials minus the averaged CS interval response to the first five CS- trials for the extinction phase; BDI-II = Beck Depression Inventory-II score; SC Mean Resp. = Skin conductance mean response to loud-tone presentations.

Stepwise logistic regression analysis identified lower estimated IQ and larger mean SC responses to the loud tones as the best combination of pre-trauma variables for predicting higher post-trauma Posterior Probability scores. The corresponding c-statistic for the Posterior Probability model derived from the maximized sample was 0.70, indicating that 70 percent of individuals with high Posterior Probability scores were correctly classified. The logistic regression analysis was repeated, adjusting for the amount of time-since-trauma and trauma-severity ratings. Odds ratios were 1.01 for the time-since-trauma variable and 1.04 for the trauma-severity variable when they were entered into the model; their contributions to the models were non-significant (Wald Chi-Square (1) = 0.04, p = .848 and Wald Chi-Square (1) = 0.05, p = .831, respectively).

## Discussion

The current study, like those of Guthrie and Bryant [40] and Pole et al. [61], represents a truly *prospective* examination of potential pre-trauma physiologic, psychologic, and demographic predictors of PTSS in firefighters and police, as predictor variables were assessed prior to the occurrence of a traumatic event rather than retrospectively. The present work contributes to findings from previous studies, which have provided indirect evidence for the pre-existing versus acquired nature of psychologic and physiologic markers associated with PTSS [31,36,38].

It is noteworthy that in the present study none of the 99 police and firefighters exposed to a traumatic event met full DSM-IV criteria for PTSD at the post-trauma assessment. The absence of clinical PTSD cases initially surprised us. When the study was originally designed we “conservatively” estimated that the PTSD rate would be about 17 % [4,62,63]. However, recent findings from prospective studies of police and firefighters suggest that a very low rate of PTSD following exposure to a traumatic event is characteristic of these populations. In their study of Australian firefighter recruits, Guthrie and Bryant [40] found that none of the 67 trauma-exposed firefighters met DSM-IV criteria for PTSD at the post-trauma assessment. Pole et al. [61] reported very low levels of PTSD symptom severities in police exposed to at least one traumatic event during the 1-year period following their initial training; only 1 of 138 police met criteria for full PTSD. Thus, the development of PTSD following exposure to a traumatic event appears to be very low in prospectively-studied samples of police and firefighters, likely lower than in the general population and perhaps reflecting a strong self-selection bias or protective benefits from professional training. One might assert that police and firefighters who are still actively engaged in their professions are simply reluctant to endorse symptoms of psychopathology. However, the fact that police and firefighters in the present study also showed normal levels of psychophysiological reactivity while recalling their traumatic events provides some objective support for the absence of significant PTSD symptoms.

The lack of police and firefighters who met full DSM-IV criteria for PTSD precluded our using the categorical diagnosis of PTSD as a post-trauma outcome and necessitated a shift in focus to pre-trauma predictors of PTSS. Despite the relatively low levels of PTSS, the combination of a higher pre-trauma depression score, lower estimated IQ and a larger differential corrugator EMG score during extinction trials of a fear-conditioning procedure significantly predicted higher PTSS. This finding accords well with accumulated evidence indicating that higher IQ appears to be a protective factor against developing PTSD [25] and the recent finding of Guthrie and Bryant [40] that a larger differential corrugator EMG response during extinction is predictive of a higher IES [64] score.

The process(es) underlying impaired extinction of corrugator EMG responses, lower IQ and higher pre-trauma depression symptoms appear to contribute to the pathogenesis of PTSS. Higher pre-trauma depression symptoms could reflect a propensity towards negative emotional affectivity [8]. Corrugator EMG activity is typically interpreted as reflecting emotional valence [65,66]. The persistence of a larger differential corrugator EMG response following fear conditioning, could reflect a pre-trauma propensity towards the *maintenance* of negative emotional responses over time. Greater intellectual ability, as reflected in IQ, likely provides more extensive cognitive resources for managing negative emotional responses [24]. Individuals with lower IQ would have more limited cognitive resources available for coping with negative emotions, which would contribute to their maintenance.

Stepwise logistic regression analysis identified the combination of lower estimated IQ and increased SC reactivity to loud tones as the best predictor of overall psychophysiologic reactivity during script-driven imagery. Guthrie and Bryant [37] and Pole et al. [61] previously reported that pre-trauma SC reactivity to loud tones (or noise) predicted subjective measures of PTSS. Their respective studies did not include a psychophysiologic outcome measure of PTSS. Taken together, findings from the studies of Guthrie and Bryant, Pole et al., and the present work suggest that pre-trauma individual differences in sympathetic nervous system reactivity, as manifest in larger SC responses to loud tones, can be used to predict the development of psychophysiologic and/or subjective PTSS. The inclusion of lower estimated IQ in the predictive model for post-trauma psychophysiologic reactivity suggests that more limited cognitive resources contribute to the maintenance of increased psychophysiological reactivity to trauma reminders, as well as contributing to the persistence of subjective PTSS.

Consideration of the SC and HR findings observed across studies using the loud-tone procedure, which provides an assessment of "unconditioned" responding, suggests that these measures may reflect different involvements of sympathetic and parasympathetic nervous system activity in the development and evolution of PTSS/PTSD. Skin conductance is primarily a measure of sympathetic nervous system activity [67], whereas HR is influenced by the parasympathetic, as well as sympathetic, nervous system. As noted above, there is accumulating evidence that increased SC reactivity to loud tones may be a pre-trauma predictor of PTSS, while there is very strong evidence that increased HR reactivity to loud tones is an acquired feature of PTSS/PTSD [e.g. [36,38]. Across studies of individuals with diagnosable PTSD, increased HR reactivity to loud tones has been reported in all but two studies, whereas increased SC reactivity has been less reliably obtained; interestingly, increased SC reactivity was observed in the two studies that failed to find increased HR reactivity (for review see [54]). This mixed pattern of SC and HR findings in loud-tone studies suggests that, whereas increased sympathetic tone/activity underlies the increased SC reactivity, it is reduced parasympathetic, and not increased sympathetic, tone that is responsible for the heightened HR reactivity to loud tones. This leads to the interesting possibility that heightened sensitivity of the sympathetic nervous system represents a risk factor for, whereas reduced parasympathetic tone is a consequence of, developing PTSS/PTSD.

The present study also observed a significant relationship between the magnitude of the HR response to CS + stimuli during conditioning and the posterior probability score. This provides some limited support for a positive relationship between pre-trauma conditionability and psychophysiologic reactivity to trauma-related imagery. The fact that pre-trauma SC reactivity to loud tones and HR reactivity to CS + stimuli during conditioning predicted post-trauma psychophysiologic reactivity to script-driven imagery but not subjective PTSS (i.e., IES-R) scores raises the possibility that these positive relationships primarily reflect relationships between measures of psychophysiologic reactivity and not with PTSS per se. However, the likelihood that the observed positive relationships simply reflect concordance amongst measures of psychophysiologic reactivity is challenged by the fact that several important measures of psychophysiologic reactivity (e.g., SC orienting response, SC and corrugator EMG responses to CS + trials during conditioning, HR and eyeblink EMG responses to loud tones) did not show relationships with the posterior probability score.

Several limitations of the reported work should be kept in mind. The relatively low, i.e., normal, level of PTSS severity raises the possibility that the relationships observed in the present study between predictor and outcome variables may not extend to clinical levels of PTSD. However, it is also possible that the reported findings underestimate the actual presence and strengths of these relationships, given the restricted ranges of scores and impact this may have had on the correlation estimates. It is possible that the extended time periods between the traumatic event and follow-up assessment for some individuals may have adversely impacted the observed relationships. Although, when the logistic regression analysis was adjusted for the amount of time elapsed between the traumatic event and assessment, the results did not change. Our preference not to correct for multiple t-test comparisons is an additional limitation and caution should be exercised when interpreting the univariate results.

Finally, although the pre-trauma differential corrugator EMG score for extinction contributed to the prediction of IES-R scores in the expected direction, this was due in part to an unexpected pattern of responses in the Low IES-R group. More specifically, the Low IES-R group produced larger corrugator responses to the CS- than to the CS+, resulting in a negative differential score. We believe that the negative mean value for the Low IES-R group is reliable, given the substantial sample size for this subgroup (n = 67); however, the reason for larger corrugator responses to the CS-, compared to CS+, is not clear. As noted above, increased corrugator activity is generally thought to reflect increased negative emotional valence. It may be that when the associative link between CS + and UCS was broken during extinction, participants in the Low IES-R suspected that the experimenter might change the associative relationship such that the CS- would predict the UCS. Why this sort of cognitive processing would be more apparent in the Low IES-R, compared to High IES-R, group is not clear. Regardless of the underlying explanation for this curious pattern of responding to the CS + and CS- during extinction, it is important to acknowledge the potential predictive value of this measure.

## Conclusions

The present study successfully identified several predictive relationships between pre-trauma psychological and psychophysiological measures and PTSS. Of particular interest, this study found that lower pre-trauma estimated IQ, a higher depression score and a larger differential corrugator EMG response following fear conditioning predicted higher IES-R scores, suggesting that the processes underlying these indices may contribute to the pathogenesis of PTSS. The combination of lower pre-trauma estimated IQ and increased SC reactivity to loud tones were found to predict greater psychophysiological reactivity (larger posterior probability score) to trauma-related cues, suggesting that processes related to these measures my contribute to the pathophysiology of PTSS. It is important to note that the levels of PTSS and general psychopathology were quite low in our sample of police and firefighters. Even so, the severity of post-trauma symptoms is dimensional and likely is normally distributed across the population. Therefore, it is possible that the predictors of PTSS identified in this study would generalize to a population with greater levels of symptomatology. It is plausible, if not likely, that the reported findings underestimate the actual presence and strengths of relationships between pre-trauma predictor variables and post-trauma outcome measures because of the limited variability in scores.

Prospective studies that identify predictors of emotional sequelae of traumatic events are particularly challenging because they necessitate extensive data collection from a sample wherein only a subset will be exposed to a traumatic event and a relatively small percentage of those exposed to trauma will ultimately develop PTSD. Despite the inherent challenges, these efforts are an important undertaking because of their potential to inform PTSD prevention and early treatment. A primary goal of prospective studies is to discover reliable markers that can aid in identifying individuals who are at highest risk for developing PTSS/PTSD and thereby allow more extensive resiliency training or early interventions to be targeted to those most likely to need them. Such interventions would be cost prohibitive if they were to be provided *en masse* and would be unnecessary for most individuals. Although significant challenges must be overcome when conducting prospective studies of PTSS/PTSD, it is our hope that future research will examine other high-risk populations, incorporate longer follow-up times and recruit larger samples. Such work would contribute substantively to the evolving literature aimed at identifying pre-trauma risk factors for PTSS/PTSD.

## Abbreviations

BDI, Beck Depression Inventory; CAPS, Clinician Administered PTSD Scale; CR, Conditioned Response; EMG, Electromyogram; HR, Heart Rate; IES-R, Impact of Event Scale-Revised; NEO-PI-R, NEO Personality Inventory-Revised; PTSD, Post-traumatic Stress Disorder; PTSS, Post-traumatic Stress Symptoms; SC, Skin Conductance; SCID, Structured Clinical Interview for DSM-IV; SCL-90-R GSI, Symptom Checklist 90-Revised; SSPQ, SCID Screen Patient Questionnaire; STAI, State-Trait Anxiety Inventory; UCR, Unconditioned Response; UCS, Unconditioned Stimulus; WAIS-R, Wechsler Adult Intelligence Scale-Revised.

## Competing interests

The authors declare no competing interests.

## Authors contributions

SPO contributed to the conception and design of the study, oversaw the conduct of the study, oversaw management and scoring of the psychophysiological data, consulted on the data analyses and drafted the manuscript. NBL conducted all diagnostic interviews and assessments and helped edit the final manuscript. MLM recruited all police and firefighter participants, served as liaison with the training academies and followed participants for the occurrence of a traumatic event. SLP contributed to the drafting and editing of the manuscript. RKP contributed to the conception and design of the study and contributed to the drafting and editing of the manuscript. YC performed the statistical analyses and contributed to drafting and editing of the Results section of the manuscript. All authors read and approved the final manuscript.
